# A vivid lesson for the whole malaria community, emphasising the Gold Standard of Education in Palestine’s malaria elimination 100 years ago

**DOI:** 10.5281/zenodo.10008064

**Published:** 2023-10-16

**Authors:** Anton Alexander

**Affiliations:** 1 BC Business Centrum, Elscot House, Arcadia Avenue, London N3 2JU, United Kingdom.

Two years ago, in 2021, I authored a paper entitled “What underscored successful malaria elimination in Palestine 100 years ago? Effective Education.” [1]. Many compliments were received. However, in view of the recent news of the killings and abductions in Israel by members of the terrorist organisation Hamas, I considered it would be useful to revisit the paper.

The following is the Abstract from the paper:

*Transmission of malaria by anopheline mosquitoes had been established by 1897, and in 1922, the first start of a successful national malaria elimination campaign began. Until then, only malaria control had been considered anywhere as a feasible project, such malaria control having been conducted primarily through larval source management. From 1922 onwards, in Palestine, by ensuring the breeding sites remained destroyed continuously over years and years, malaria elimination was eventually achieved. However, in order to achieve such continuous destruction, transmission of the disease had to be imaginatively and sensitively explained to all the inhabitants who thereupon willingly accepted the task of ensuring the breeding sites remained destroyed. Without that education, the inhabitants would not have provided the continuous work required, and Palestine would have remained in its severe malarious state.*

The essence of the paper was to explain the importance of education in malaria elimination, to ensure inhabitants understood and therefore appreciated why certain anti-mosquito activities were necessary and why it was a necessity for mosquito breeding sites to remain destroyed. a major component in malaria elimination. Unfortunately, today this component appears often to be the subject of lip-service and without genuine attention being paid to it.

It has been suggested that education and community engagement represent 80% of the work and attention in malaria elimination. It has been helpful for education/community engagement to be coloured in this way in order to give weight to it as

But the world within this current month of October 2023 has just been witness to a depravity which plumbs new depths of evil. The world has had presented to it images of killing of Jews because they were Jews on a scale unseen since World War II that ended in 1945. The malaria community must not forget the shock it first felt when the news of this event first broke, and the community must try to imagine the fear experienced by the inhabitants of the towns and settlements in Israel alongside Gaza as the Hamas gunmen roamed these locations seeking out people to kill.

The 2021 paper stressed the Arab/Jew cooperation in the anti-malaria works as a result of the education 100 years ago. Also featured in the paper was the attempt to disrupt that cooperation by the Mufti of Jerusalem, a rabid anti-semite, the head of the Muslim Brotherhood in Palestine, and whose organisation was the forerunner of Hamas today. Yet the Arab/Jew cooperation 100 years ago was strong and endured despite the Mufti’s attempt to disrupt it. The Mufti failed to disrupt the cooperation because the education of the inhabitants had been so successful, the inhabitants had understood and appreciated why the anti-mosquito tasks were necessary.

To live a life free from malaria had become a priority.

The recent gruesome Hamas killings and abductions of Jews were indeed reminiscent of the fate of Jews during World War II at the hands of the Nazis. With the recent conduct and behaviour of Hamas mirroring the conduct and behaviour of the Nazis during World War II, it is therefore unsurprising to read in the 2021 paper that when the Mufti’s violence became unacceptable to the British Mandate, he managed to escape arrest and eventually fled to Nazi Germany, where he assisted Hitler with his war efforts.

The intention of this paper is to have the reader try to relate to and understand the enormous achievement of malaria elimination in Palestine 100 years ago. The reader is called upon to imagine the educational level which caused malaria elimination to become a priority, and which incidentally happened to make the inhabitants so committed to the idea of malaria elimination as to enable them to also withstand the Mufti’s violence, fears and threats. That education was a Gold Standard. It is true that such a standard was then more readily possible because of the very small Palestinian population, which enabled personal education, even one-on-one education if necessary, of both of Arabs and Jews. It serves to demonstrate that such a level of education can exist. Also, it is useful to point out what an aid it must have been for conducting any anti-malaria work to have the assistance of such enthusiastic inhabitants.

The Mufti’s violence was severe against Jews and also anyone else who worked with the British Mandate or with the Jews. Many Arabs who didn’t agree with, or who wouldn’t support the Mufti were also targeted and obliged to flee and leave Palestine. Because so many Arabs fled, the total population (namely the total of both Arabs and Jews) in Palestine in 1938 was in fact less than the total in 1937.

Despite this backdrop of violence, because malaria elimination had always been a priority, education also remained a priority. Education was recognised at that time as being absolutely essential for malaria elimination to be successful.

The 2021 paper concluded the account of the Mufti’s attempted disruption with the following: Sadly, the Mufti’s violence and indifference to the harmful consequence of his conduct on the Arab inhabitants has travelled down time. To assist the reader in appreciating the level of violence which Kligler’s cooperation had to withstand, the reader should try to examine the organisation, Hamas, which rules Gaza today, and its reported terror and violence which appears to be replicated from the Mufti’s own violence and terror. This is unsurprising as Hamas (classified as a violent terrorist organisation by many countries) is a branch of the same anti-Semitic Muslim Brotherhood that was headed by the Mufti. Yet Kligler managed to create cooperation, between Jews and Arabs, from such unlikely raw material; evidence of the strength and effectiveness of Kligler’s education. Kligler, for those not familiar with his work and accomplishments, was the architect 100 years ago of the Palestine malaria elimination referred to above.

Despite the violence, the education and the resulting cooperation remained intact within Palestine all those years ago. The news of the Hamas violence can now provide a feeling of the weight, impact or significance of education which must have been provided to enable the Palestine cooperation 100 years ago to survive, despite the Mufti’s violence and threats. This can now assist when considering provision of an adequate level of education for future malaria elimination campaigns if such campaigns are to be successful around the world. The intention of this paper is also to cause the reader to focus and to try to appreciate before-hand what steps with each separate community need to be taken when seriously considering malaria elimination. Sometimes even individual education may be necessary because it should not be forgotten that communities are in fact made up of individuals. If a reader is not treating malaria elimination in this way, then he/she is not taking malaria elimination seriously.

Education is not some secondary or minor step, and ideally, it should theoretically be truly effective enough to withstand the type of conduct we have witnessed from Hamas. As an emotion, for the education to be effective, all inhabitants individually must feel the need for malaria to be eliminated. It is not good enough merely telling the inhabitants they should be rid of the disease – they have to need to be rid of it. They have to genuinely want to be free from it (a suggestion when considering the problem is to equate it with giving up smoking, or losing weight). Only then will the malaria community receive the full cooperation of the inhabitants and only then will malaria be eliminated. And that will only be achieved with proper education, no matter how long that education takes to be achieved.
